# *In ovo* administration of human recombinant leptin shows dose dependent angiogenic effect on chicken chorioallantoic membrane

**DOI:** 10.1186/s40659-015-0021-z

**Published:** 2015-06-10

**Authors:** Reji Manjunathan, Malathi Ragunathan

**Affiliations:** Department of Genetics, Dr. ALM PG IBMS, Taramani Campus, University of Madras, Chennai 600 113, Tamilnadu, India

**Keywords:** Angiogenesis, HRL, Human Recombinant Leptin, CAM, chorioallantoic membrane, ECs, endothelial cells, VEGF, vascular endothelial growth factor, MMP, matrix metalloproteinase, Blood vessels, CD34

## Abstract

**Background:**

Leptin, the cytokine produced by white adipose tissue is known to regulate food energy homeostasis through its hypothalamic receptor. *In vitro* studies have demonstrated that leptin plays a major role in angiogenesis through binding to the receptor Ob-R present on ECs by stimulating and initiating new capillary like structures from ECs. Various *in vivo* studies indicate that leptin has diverse effect on angiogenesis. A few reports have showed that leptin exerts pro angiogenic effects while some suggested that it has antiangiogenic potential. It is theoretically highly important to understand the effect of leptin on angiogenesis to use as a therapeutic molecule in various angiogenesis related pathological conditions. Chicken chorio allantoic membrane (CAM) on 9th day of incubation was incubated with 1, 3 and 5 μg concentration of HRL for 72 h using gelatin sponge. Images where taken after every 24 h of incubation and analysed with *Angioguant* software. The treated area was observed under microscope and histological evaluation was performed for the same. Tissue thickness was calculated morphometrically from haematoxylin and eosin stained cross sections. Reverse transcriptase PCR and immunohistochemistry were also performed to study the gene and protein level expression of angiogenic molecules.

**Results:**

HRL has the ability to induce new vessel formation at the treated area and growth of the newly formed vessels and cellular morphological changes occur in a dose dependent manner. Increase in the tissue thickness at the treated area is suggestive of initiation of new capillary like structures. Elevated mRNA and protein level expression of VEGF165 and MMP2 along with the activation of ECs as demonstrated by the presence of CD34 expression supports the neovascularization potential of HRL.

**Conclusion:**

Angiogenic potential of HRL depends on the concentration and time of incubation and is involved in the activation of ECs along with the major interaction of VEGF 165 and MMP2. It is also observed that 3 μg of HRL exhibits maximum angiogenic potential at 72 h of incubation. Thus our data suggest that dose dependent angiogenic potential HRL could provide a novel role in angiogenic dependent therapeutics such as ischemia and wound healing conditions.

## Background

The *Ob*-gene product leptin is 16 kDa peptide hormone, produced mainly by white adipose tissue is proposed to play a key role in the regulation of body weight and thermogenesis through the receptor found in the hypothalamus [1]. Accumulating evidences from *in vitro* and *in vivo* assays suggest that leptin promote endothelial cell (EC) proliferation and survival in favour of angiogenesis [2, 3]. *In vivo* and *in vitro* results from Hyung *et al.* study indicates that angiogenic potential of leptin is mediated by matrix metalloproteinase (MMPs) [4]. However, the actual mechanism behind the involvement of matrix metalloproteinase in leptin mediated angiogenesis is not clear.

The effect of leptin on angiogenesis is still not understood as some reports have suggested its potential to induce angiogenesis while some have indicated of its anti angiogenic effect [5–9]. Recently it has been reported that *in ovo* administration of leptin inhibited angiogenesis on chicken chorio allantoic membrane (CAM) [10]. Leptin has been administered as a therapeutic molecule in various angiogenesis related pathological conditions especially in wound healing [11]. However a quantitative *in vivo* evaluation on the effect of leptin on angiogenesis is important because of its therapeutic application potential in the pathology of angiogenesis dependent conditions. Therefore in the present study we examined the angiogenic potential of human recombinant leptin (HRL) using well vascularized CAM of developing chicken embryo (G*allus gallus domesticus*). The advantage of CAM assay is that it is a highly vascularized structure with potential growth and this *in ovo vivo* system is highly useful to understand the physiological angiogenesis and hence widely used for the screening of various compounds for their angiogenic activity [12].

In the present study we analysed and compared the angiogenic ability of HRL of varying concentrations such as 1, 3 and 5 μg for an incubation period of 72 h using late CAM. Gelatin sponges soaked with leptin were placed on the membrane at 9th day of post incubation so as to allow slow delivery of the chemical at the treated area with less or no inflammation [13, 14]. The ability of HRL to induce new vessel growth at the treated area is directly visualized from the CAM images taken at different time period of incubation and growth of these vessels measured with *Image J* and *Angioquant* MATLAB softwares [14–16]. Angiogenic response of HRL at the treated area is further analysed in detail from the histological sections. We also examined the expression of major angiogenic molecules at the molecular and protein level to understand the involvement of these factors on HRL induced angiogenesis. Our findings suggest that the potential of HRL to induce angiogenesis depends on various physiological factors especially dose and time of incubation. The result have demonstrated that HRL favours neovascularization through sprouting of vessels which is accelerated by the expression of VEGF165 and MMP2 in a dose dependent way in chicken CAM vasculature.

## Methods

### Materials

Fertilized white leghorn chicken eggs were purchased from Tamil Nadu Poultry Research Station, Madras Veterinary University, Nandanam, Tamil Nadu, India. Gelatin sponges purchased from Jhonson & Jhonson Pvt Lmtd, India, Paraffin film and wax were purchased from Sigma, Aldrich, USA. Haematoxylin and Eosin stain purchased from Medox, India and Human recombinant leptin from MP Biomedicals, Inc. France. TRI*zol* reagent, agarose and EtBr were from Sigma, Aldrich, USA. ImProm-11™ Reverse Transcriptase kit and GoTaq Green Master Mix PCR amplification kit were from Promega, USA, Oligo (dt) of length 18-meres from eurofins, mwg operon, Germany, Random hexamers from MP Biomedicals, USA. All primers were purchased from Bioserve, India. DNA ladders purchased from Invitrogen, USA. DAB system purchased from Bangalore Genei, India. CD34 antibody (Endothelial Cell Marker, Cluster designation 34) from US Biological, USA and VEGF A from CALBIOCHEM, EMD. Bradford reagent and FITC (Goat ant-rabbit IgG were from Bangalore Genei, India. SDS-PAGE Standards marker was from BIO-RAD, CANADA and gelatin from Medox, India. Rabbit polyclonal MMP2 is a kind gift from Dr. Li Haiqing, MD, Ph.D, −Technology transfer specialist, National Cancer Institute, Rockville, USA. Unless otherwise specified all other common reagents and chemicals were purchased from Sigma, Aldrich, USA.

### The *in ovo* CAM model

Fertilized white leghorn chicken eggs weighing 50 ± 2 g, were incubated at 37 °C in a ‘humidified atmosphere (>60 % relative humidity) as per the protocol described in Hen’s Egg Test - Chorioallantoic Membrane (HET-CAM) method adapted from ZEBET (The German Centre for the Documentation and validation of Alternative Methods, Republic of Germany). At day 3 of post incubation, 2 to 3 ml of albumin was withdrawn, using a 21 gauge needle, through the large blunt edge of the egg in order to minimize the adhesion of the shell membrane with CAM. A square window of 2 cm^2^ was opened in the egg shell and sealed with paraffin film to prevent dehydration and the eggs were incubated further. At day 9 of post incubation, gelatin sponges of size of 1 mm^3^ were placed on top of the growing CAM under sterile condition [14, 17] and were soaked with 15 μl volume of 1, 3 and 5 μg concentration of HRL. Control eggs were incubated with 1X PBS (pH-7.3). The window was closed with a transparent adhesive tape and eggs were incubated for 72 h until it reached post incubation day 12. CAM were photographed at 0, 24, 48 and 72 h using Canon digital camera and images were analysed with *Image J* and *Angioquant* Toolbox, MATLAB 6.5 software to measure total length and size of the blood vessels (micrometre) from the area of treatment.

### Light microscope analysis

After 72 h of incubation the area of the CAM treated with HRL was detached carefully. The excised membrane was kept on glass slides and images were taken using light microscope both at 4 and 10× magnification to view the growth of capillaries [18].

### Histology

After 72 h of incubation area of the CAM treated with HRL was flooded with Bouin’s fixative solution. Around 1 cm^2^ of the membrane around the treated area was removed carefully using forceps and surgical scissors and dehydrated through graded series of alcohol (50 %, 70 %, 90 % and absolute) and embedded in paraffin wax. Vertical cross tissue sections (7 μm in thickness) were taken using Rotary Microtome (Weswicox, Japan). Sections were treated with alcohol in ascending order (absolute, 90 %, 70 %, and 50 %) and cleared with xylene before staining with haematoxylin and eosin. After mounting with DPX, the histological sections were observed under light microscope at 40× magnification for qualitative assessment and images were recorded using Nikon Camera attached with light microscope at 10× magnification [14].

### Morphometric analysis of CAM tissue thickness (D*CAM*)

Thickness of the CAM for all the groups treated with HRL were measured from haematoxylin and eosin stained vertical cross sections using a calibrated objective at 40× magnification with 10 × 10 calibrated grid at 10× ocular. Distances between the chorionic and allantoic epithelial layers were measured in micrometre at 6 different locations from the same sample and is repeated for six serial cross sections of the same. Average tissue thickness was calculated from each tissue sample of the same and obtained a mean D*CAM* thickness [14, 19].

### Semi-Quantitative Reverse Transcriptase–Polymerase Chain Reaction (RT-PCR)

Total RNA was isolated from CAM treated with HRL using TRI*zol* reagent (100 mg/1 ml). The quantity and the purity of the isolated RNA were checked using UV-visible spectrophotometer. cDNA of 20 μl volume was synthesized using ImProm-11™ Reverse Transcriptase kit with Oligo (dt) of length 18-meres and random hexamers. PCR amplification was performed using GoTaq Green Master Mix kit and changes in the level of mRNA expression of VEGF165 [20], VEGF121 [21], MMP2 [20], MMP9 [22] and GAPDH [23] were evaluated using PCR with 100 pico moles of specific primers. The relative expression level of each mRNA transcript was normalized with that of control. PCR products were subjected to electrophoresis.

### Gelatin zymography

CAM tissues at the treated area from each group were homogenized (100 mg/ml) using Tris buffer (0.5 M Tris–HCl (pH-6.8), 10 % SDS, glycerol and 0.01 % bromophenol blue) and centrifuged at 12,000 rpm/4 °C/10 min. The concentration of protein from the supernatant was determined using Bradford reagent and the gelatinase activity was examined on a 10 % SDS-PAGE electrophoresis containing 1.0 mg/ml of gelatin. Protein samples of 25 μg/40 μl were loaded per well along with 20 μl pertained SDS-PAGE Standards marker. After electrophoresis, the gels were washed with 2.5 % of Triton-X-100 and incubated in digestion buffer (50 mM Tris HCl-pH-7.5,100 mM CaCl2, 1 μM ZnCl2, 1 % Triton X-100, 0.02 % NaN3-100 ml) for 16 to 18 h at 37 °C with gentle agitation. The gel was stained with staining solution (0.05 % (*w/v,* Coomassie blue in 50 % methanol and 10 % acetic acid) for 1 h and de stained with methanol/acetic acid mixture. Gelatinase activity of MMP2 was detected as clear white bands against background. The gels were scanned and images were recorded using BIO-RAD Calibrated Densitometer Software (GS 800, USA). The density of the bands was calculated with PD Quest Advances Software and normalized with control value [24].

### Immunohistochemistry

Three different concentration namely 1, 3 and 5 μg of HRL have been used of which 3 μg of HRL yielded maximum angiogenic response when compared to other two concentrations. The deparafinized and dehydrated CAM of 5 μM thickness after treating with 3 μg of HRL was allowed to undergo antigen retrieval process using Sodium Citrate (10 mM-pH 6.0) in a microwave oven for 20 min followed by washing in DDH2O for 3X5 min in 1X PBS (pH 7.3). Normal Goat Serum Blocking Solution (2 % goat serum,1 % BSA, 0.1 % cold fish skin gelatin, 0.1 % Triton X-100, 0.05 % Tween- 20, 0.05 % Sodium Azide, 0.01 M PBS (pH 7.2) of 50 to 75 μl was added immediately on the sections and incubated for 1 h in a humidified chamber. After washing with 1X PBS, primary antibodies of MMP2 (1:200), VEGF (VEGF A) (1:100) and CD34 (1:200) diluted in blocking serum were applied on the sections and after overnight incubation rinsed with 1X PBS with 0.05 % of Tween-20. Diluted FITC (Goat ant-rabbit IgG) and HRP (both Goat anti-rabbit and Goat anti- mouse IgG) secondary antibodies of 1:40 dilution was applied for 1 h according to manufacturer’s instruction. For HRP conjugated secondary antibodies DAB system was used for colour development. The slides were finally counterstained with Mayer’s haematoxylin and mounted with 90 % of glycerol. For HRP conjugated system the images were recorded using light microscope and for FITC conjugation BX51 Olympus Fluorescence Microscope at a wavelength of 515 nm with ASI FISH View 5.5 software at 40× magnification [25].

### Data analysis and statistical analysis

All experiments were independently performed in triplicate. Data were analysed using one way *ANOVA* analysis of variance test and *Tukey* post hoc test as appropriate (Sigma stat 2.0). Data were expressed as means + S.E.M and P-values of *p = < 0.001 and #p = 0.001 were selected for showing statistically significant difference.

## Results

### Human recombinant leptin induces neo vascularization - visual assessment of new blood vessel formation on CAM vascular bed and its growth

**Fig. 1:**
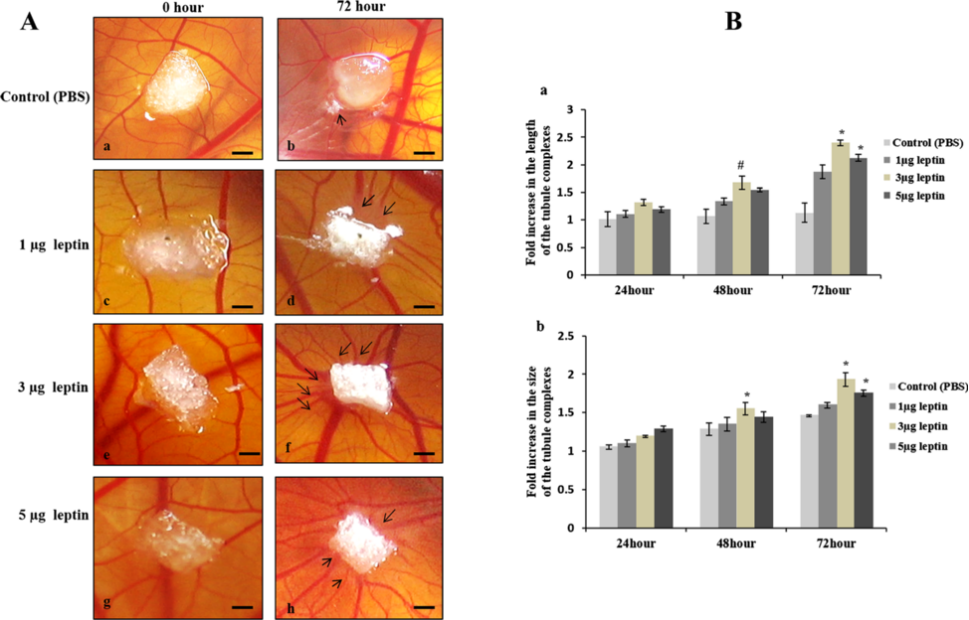
Human recombinant leptin initiate new blood vessel formation and accelerate vessel growth. **a** Images of CAM treated with 1, 3 and 5 μg concentration of HRL and control with 1X PBS for 72 hours. Figure panel represents the images recorded at 0 and 72 hours of incubation. Increased blood vessels formation around the sponge as spoke wheel pattern was observed in CAM treated with 3 and 5 μg of HRL (**f** and **h**) than 1 μg treated CAM when compared to control (**b**) at 72 hours of incubation. Figure **a**, **c**, **e** and **g** represents 0 hour images of CAM for control , 1 and 3 and 5 μg of HRL respectively. Images are taken using Cannon digital camera at 4X magnification and bar is 10 μM. Images are representative of 3 set of experiments. Arrow indicates the presence of new blood vessels. **b** Images of the CAM recorded at 0, 24, 48 and 72 h of incubation were analysed with Image J and *Angioquant* software to measure total vessel length (graph **a**) and vessel size (graph **b**) of the capillaries. CAM treated with 3 μg of HRL shows significant increase in the length and size of the blood vessels at 48 h of incubation. At 72 h of incubation both 3 and 5 μg of HRL shows significant increase in the vessel length and size. Though both the concentration shows significant increase, the angiogenic potential of 3 μg of HRL is comparatively higher than that of 5 μg of HRL. Value at 0 h was taken as one. Experiments were performed in triplicate and data presented as mean + SEM, (*p = <0.001 and #p = 0.001 versus control)

The main focus of the present study is to visualize directly the angiogenic ability of HRL to induce new vessel formation at the treated area. CAM incubated with all three concentrations of HRL shows growth of new capillary vessels at the treated area (Fig. 1a). After 72 h of incubation, 1 μg of HRL (Fig. 1a. d) exhibited minimal growth of capillaries around the sponge and is visually similar to that of control CAM (Fig. 1a. b). Formations of new capillaries were more prominent with 3 μg of HRL after 72 h and this is evident with development of reddishness around the sponge indicating increased vessel growth at the treated area (Fig. 1a. f). Spoke wheel pattern of allantoic vessel growth is observed for this particular concentration. HRL of 5 μg also shows numerous scattered allantoic vessels in and around sponge at 72 h but with lesser vessels growth when compared to that of 3 μg (Fig. 1a. h).

From the above observation it can be inferred that, both 3 and 5 μg concentration of HRL has the ability to induce angiogenesis through new capillary formation at 72 h of incubation. In order to conform this and also to arrive at the optimum working concentration for HRL we measured the growth of the vessels in terms of its length and size, from the images taken at 0, 24, 48 and 72 h of incubation with *Image J* and *Angioquant* MATLAB software. This software is specially made for quantifying angiogenesis using *in vitro* assays and has been employed for CAM with some modifications using *Image J* software [11, 13]. Of the three different concentrations, used 3 μg of HRL demonstrates a significant increase in the vessel length (Fig. 1b. b) and size (Fig. 1b. b) at 48 h of incubation which is 1.99 fo1d greater than that of control (#p = 0.001, *p = <0.001). At 72 h of incubation both 3 and 5 μg of HRL induces a significant increase in the vessel length and size (*p = <0.001) and of these two, 3 μg of leptin shows 2.1 fold increases in vessel growth which is higher than that of 5 μg that showed which has only 1.8 fold increase. HRL of 1 μg also shows angiogenic potential with increase in the vessel growth up to 1.5 fold, the value is not significant when compared to control that has 1.2 fold increase in the vessel growth from 0 to 72 h of incubation. Thus it is obvious that HRL can induce new vessel formation at the treated area in a dose dependent manner with 3 μg concentration having maximum angiogenic response.

### Human recombinant leptin initiate growth of vessels by means of sprouting – visualising the morphology of blood vessels on CAM vascular bed

**Fig. 2:**
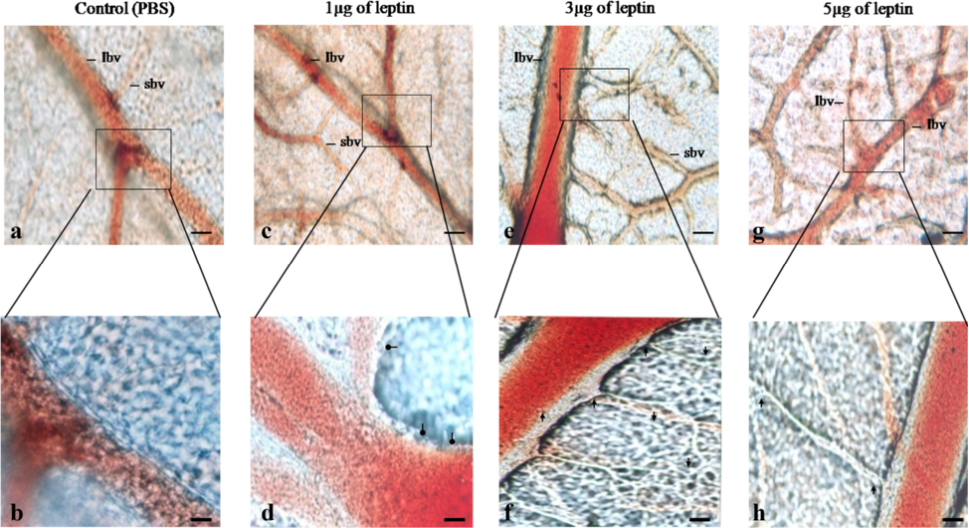
Human recombinant leptin able initiate sprouting of new vessels from existing one. Light microscopic images of CAM treated with 1, 3 and 5 μg of HRL and control with 1X PBS for 72 h (Figures **b**, **d**, **f**, **h** are the enlarged version of **a**, **c**, **e**, **g** respectively). Images of CAM treated with 3 and 5 μg of HRL (**e**, **f**, **g** and **h**) shows the presences of branches and sub branches from the main blood vessels as an indication of new blood vessel formation by sprouting and CAM treated with 1 μg of HRL (**c** and **d**) shows the presence of bulging like structures as an indication of beginning of sprouting of blood vessels when compared to control (**a** and **b**). Among 3 and 5 μg of HRL 3 μg shows the presences of more branches and sub branches at the treated area. Figure represents the images recorded at 72 h of incubation using light microscope at 20X magnification of bar is 20 μM and are representative of 3 set of experiments. Arrow indicates the presence of branches and sub branches from the main vessel

We analysed the morphology of blood vessels in order to confirm the sprouting angiogenic ability of HRL at the treated area. Images of the CAMs were recorded immediately after 72 h of incubation using microscope. It was found that HRL is able to induce growth of new blood vessels from the existing one (Fig. 2). CAM incubated with 3 μg of HRL shows more number of sprouting vessels along with branches and sub branches at the treated area (Fig. 2e). An enlarged view of the same treated area shows the presence of more branches form the main vessel and sub branches from the newly formed vessels, a feature directly suggesting the fact that HRL of this particular concentration and time of incubation exerts its maximum angiogenic activity (Fig. 2f). CAM treated with 5 μg of HRL also shows the presence of sprouting of vessels from the main one with appearances of branches and sub branches (Fig. 2g and h), but its potential to induce new vessel formation at the treated area is comparatively lesser than that of 3 μg HRL. HRL of 1 μg also shows the presence of sprouting structures from the main vessel, but is not remarkably strong when compared to other concentrations (Fig. 2c). An enlarged view of the treated area for the same shows roughness throughout the outer surface of the main blood vessel along with bulging appearance suggestive of its potential to initiate sprouting of new blood vessels at the treated area. Control CAM doesn’t show any such sprout formation from the existing vessels (Fig. 2a) and the enlarged view of the same indicates stable blood vessel without any rough appearance or bud like structures from that of main one (Fig. 2b). Thus, HRL is able to induce sprouting of new vessels from the existing one with maximum sprouting efficacy at 3 μg concentration.

### Human recombinant leptin induces morphological changes leading to neo vascularization – histological observation of the CAM vasculature

**Fig. 3:**
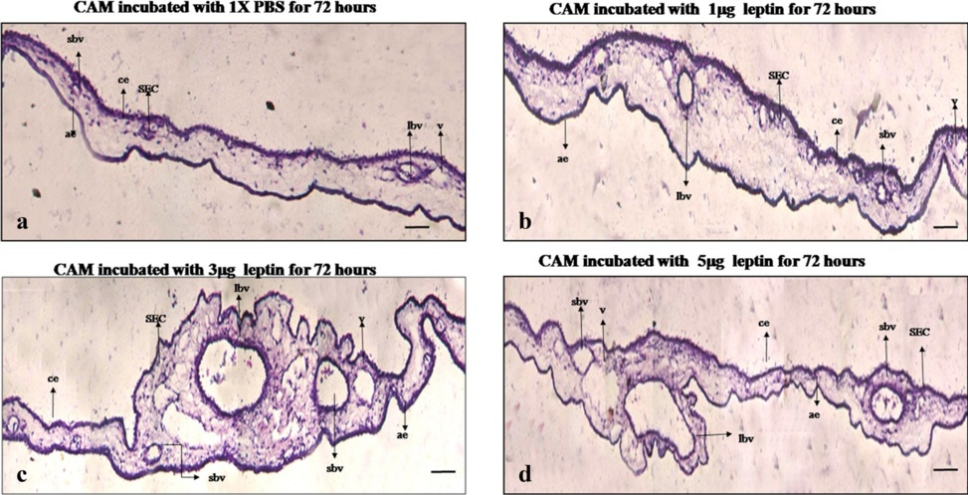
Human recombinant leptin induces morphological changes in favour of angiogenesis. Haematoxylin and eosin stained histological images of CAM treated with 1, 3 and 5 μg of HRL and control with 1X PBS for 72 h. Control CAM (**a**) shows thin chorionic (ce) and allantoic (ae) epithelial layers with sub epithelial capillary network (SEC), CAM treated with 1 μg of HRL (**b**) shows comparatively thicker chorionic and allantoic epithelial layer along with small vessels at the stroma region. CAM treated with 3 μg of HRL (**c**) shows the presences of numerous large vessels at the surrounding of large one with increased tissue thickness at the primary stratum. CAM treated with 5 μg of HRL (**d**) comparatively lesser number of small vessels around the large one than CAM treated with 3 μg of HRL. Gelatin sponges has not caused wound or inflammation. Representative sections are 7 μM in thickness and were recorded at 10X magnification and bar is 50 μM. (v for vein, lbv for large blood vessel and sbv for small blood vessel)

To conform the angiogenic potential of HRL, we also analysed the changes in the cellular morphology of the CAM vasculature from cross sections of the treated area stained with haematoxylin and eosin. Changes in the morphology of the CAM vasculature has been changed observed for all three concentrations of HRL and is indicative of the positive angiogenic response due to HRL (Fig. 3). CAM incubated with 1 μg of HRL (Fig. 3b) shows irregular appearance of thin stratum (which contains both chorionic (ce) and allantoic (ae) epithelial layers) with the presences of few blood vessels at the primary stratum. CAM treated with 3 μg of HRL (Fig. 3c) shows vessels enlarged in size with remarkable irregular growth pattern at thin stratum. Morphology of ECs are altered those ECs which are present at the capillary plexus/blood sinus area (which lies in between the primary and thin stratum) shows sprouting appearance typical for angiogenesis. Thickness of the primary stratum is also increased probably due to cellular accumulation at the sub epithelial capillary network (SEC). Presences of a number of small blood vessels around the main vessels is suggestive of the angiogenic potential of leptin either by forming new vessels or by inducing the sprouting from the main one. CAM treated with 5 μg of HRL (Fig. 3d) also shows irregular appearances at thin stratum due to increase in the number of blood vessels and diminished accumulation of cells were observed beneath SEC. Very few small vessels were found around the large one as an indication of minimal sprouting which is lesser than that observed with 3 μg. Control CAM (Fig. 3a) shows a few scattered blood vessels at primary stratum with uniform growth at the thin stratum due to growth of less number of blood vessels at this area. These observations demonstrates that HRL is able to induce formation of new vessel growth by inducing positive morphological changes at the cellular level in favour of angiogenesis. Leptin of 3 μg concentration is capable of eliciting promising cellular response that could lead to more angiogenic growth.

### Human recombinant leptin increases the size of tissue by means of inducing new vessel growth. Quantitative measurement of the angiogenic potential of human recombinant leptin by morphometric measurement of CAM tissue thickness (D*CAM*)

**Fig. 4:**
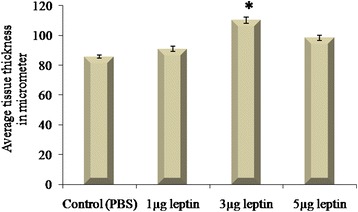
Human recombinant leptin increases the size of tissue by means of inducing new vessel growth. Total thickness of the tissue at the treated area with 1, 3 and 5 μg of HRL was measured from haematoxylin and eosin stained vertical cross section after 72 h. Control CAM was treated with 1X PBS. The distance between chorionic and allantoic epithelial layers was calculated morphometrically in μM. CAM treated with 3 μg of HRL shows significantly increased tissue thickness than other two concentrations. Material shrinkage is estimated to be ~25 % relative to the fresh material in all cases. Experiments were performed in triplicate and data presented as mean + SEM, (*p = <0.001versus control)

The ability of 1, 3 and 5 μg of HRL to induce new vessels growth was further confirmed by measuring the size of the tissue from the from haematoxylin and eosin stained paraffin embedded vertical cross sections and is plotted as average tissue thickness (Fig. 4). Formation of new blood vessels or enlargement or growth of the existing blood vessels will cause an increase in the thickness of the CAM at the stroma and will push both the chorionic and allantoic epithelial layer a part. In the present study we calculated the distance between these two layers (thin stratum) morphometrically in μM after 72 h of incubation. It is found that thickness of the CAM is considerably increased significantly for 3 μg of HRL (*p = <0.001) when compared to other two studied concentrations suggestive of its ability of to induce angiogenesis under this concentration. In paraffin-embedded tissues, material shrinkage is estimated to be ~25 % relative to the fresh material and since all tissues were prepared similarly, tissue shrinkage is assumed to be same for all CAM zones. Thus, shrinkage corrections are not necessary while comparing tissue thickness [26].

### Angiogenic potential of human recombinant leptin depends on the activation of major angiogenic growth factors - molecular profiling of VEGF 165, VEGF 121, bFGF2, MMP2 and MMP9

**Fig. 5:**
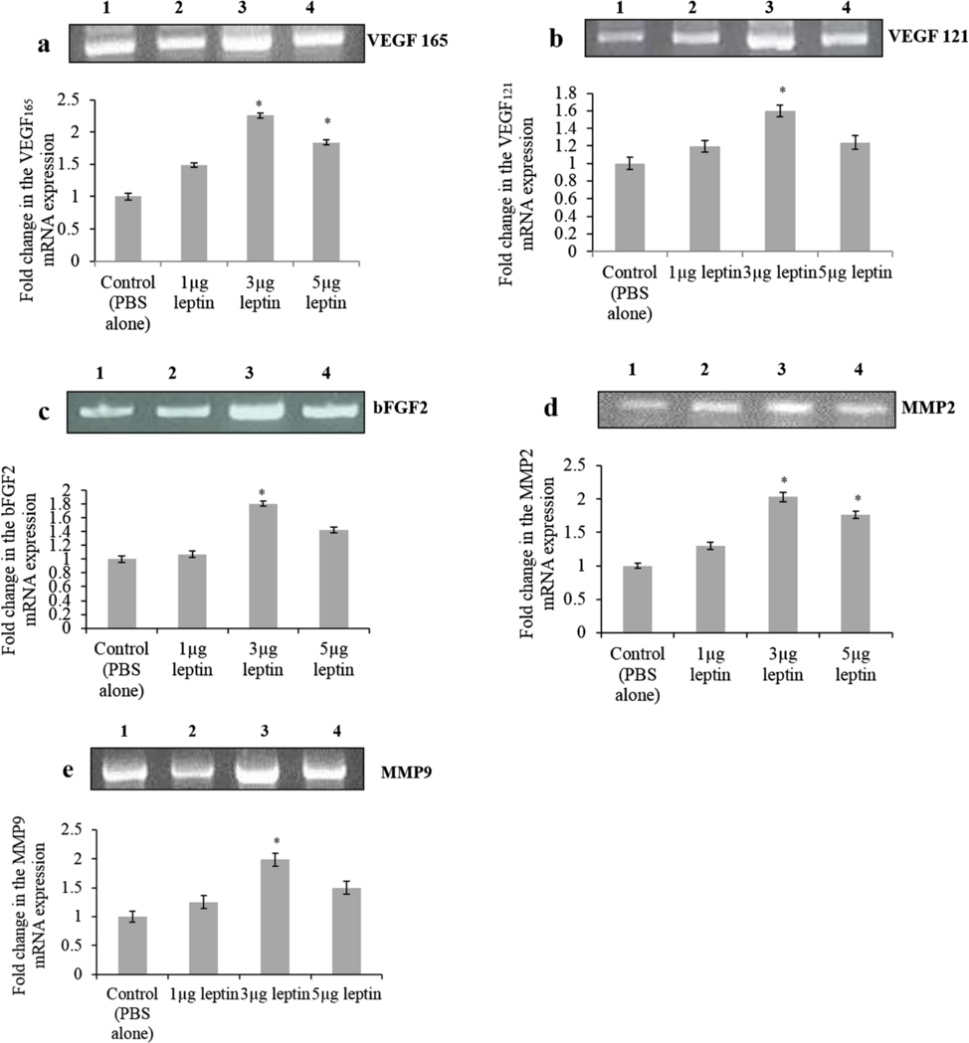
Human recombinant leptin enhances the mRNA level expression of VEGF165, VEGF121, bFGF2, MMP2 and MMP9. Images of Reverse Transcriptase- PCR products of (**a**) VEGF165 (**b**) VEGF121 (**c**) bFGF2 (**d**) MMP2 and (**e**) MMP9 from CAM after treated with 1, 3 and 5 μg of HRL and control with 1X PBS for 72 h. GAPDH was used as internal loading control. The transcripts were confirmed by 100bp DNA ladder. Graph represents the OD value ratio of mRNA transcripts after normalizing with GADPH OD value of the same. The OD value was measured using *Image J* software. The relative level expression of specified genes were increased significantly for 3 μg of HRL and under 5 μg of HRL the relative expression of VEGF 165 and MMP2 increased significantly after 72 h of incubation.. Each value is the mean ± SEM, *p= <0.001 versus control, n = 3. Experiments were performed in triplicate and data presented as mean + SEM. (Lane 1 - control, lane 2 – 1 μg HRL, lane 3 – 3 μg HRL and lane 4- 5 μg HRL)

The expression of primary angiogenic growth factors such as VEGF, bFGF2, MMP2 and MMP9 during leptin induced angiogenesis is analysed by measuring the variation in the mRNA level. In our study it is observed that mRNA level expression of these specified angiogenic growth factors increased when treated with HRL (Fig. 5). The intensity of the bands were measured as relative OD and given in Fig. 5b. There was a consistent increase in the expression pattern for 3 μg HRL which is significantly higher (*p = <0.001) than that of other two concentrations. It was also observed that for 5 μg of HRL the relative mRNA level of VEGF165 and MMP2 increased significantly (*p = <0.001) owing to the involvement of these two main angiogenic growth factors on HRL induced angiogenesis. The gene profiling data indicates that the angiogenic ability of HRL mainly depends on the activation VEGF165 and MMP2 since the expression value have increased significantly when treated with 3 and 5 μg of HRL. Thus, HRL at 3 μg concentration is able to induce maximum angiogenic growth by increasing the gene expression of VEGF121, bFGF2, MMP9 along with VGEF165 and MMP2.

### Human recombinant leptin accelerates the gelatinase activity of MMP2

**Fig. 6:**
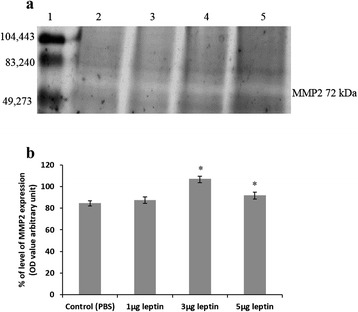
Human recombinant leptin enhances the gelatinase activity of MMP2. **a** Image represents the gelatin zymography of MMP2 activity from CAM after treated with 1, 3 and 5 μg of HRL and control with 1X PBS for 72 h. The image shows that the gelatinase activity was increased under 3 and 5 μg of HRL which is visible as white band against black background. Image is the result of 3 set of experiments. **b** MMP2 gelatinase activity was measured as OD value using *Image J* software. Percentage of MMP2 gelatinase activity increased significantly under 3 and 5 μg of HRL with higher percentage under 3 μg of HRL. Each value is the mean ± SEM, *p = <0.001 versus control, n = 3. (Lane 1- protein weight marker, Lane 2 - control, lane 3–1 μg HRL, lane 4–3 μg HRL and lane 5–5 μg HRL)

The potential of HRL to induce neo vascularization was measured intern by its ability to accelerate gelatinase activity of MMP2 to degrade ECM in order to favour ECs proliferation and migration. Figure 6a indicates that the gelatinase activity of MMP2 is increased indicating ECs proliferation and activation when treated with HRL. The intensity value measured in terms of OD and the graph plotted (Fig. 6b) shows that the gelatinase activity of MMP2 is increased significantly (*p = <0.001) when treated with 3 and 5 μg of HRL. The gelatin zymogram analysis of MMP2 activity demonstrates that HRL has the potential to accelerate neo vascularization by means of increasing the degradation of ECM. This data also indicates that irrespective of the concentration, HRL is able to accelerate the gelatinase activity of MMP2 which in turn signifies that MMP2 plays a major role in HRL induced angiogenesis.

### Human recombinant leptin enhances activation of endothelial cells

**Fig. 7:**
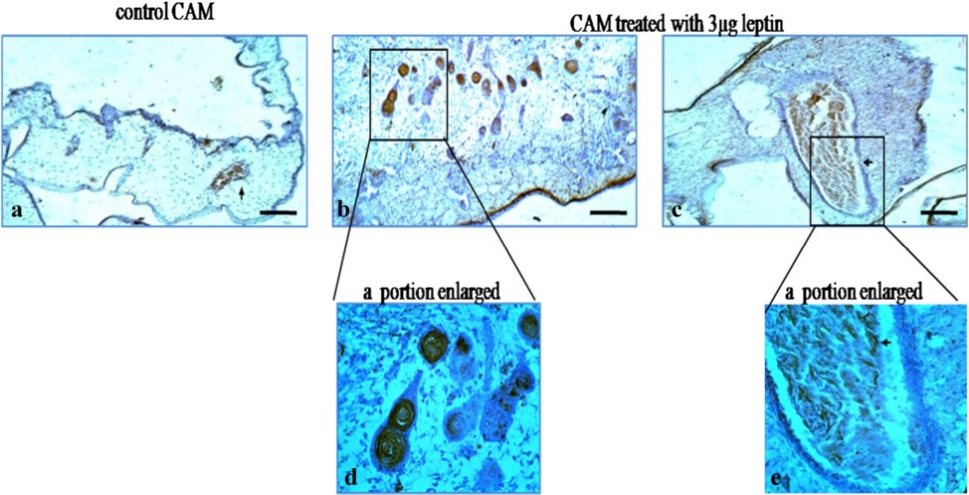
Human recombinant leptin activate endothelial cells. Images of CAM stained for CD34 under 3 μg leptin for 72 h. Control CAM treated with 1X PBS for 72 h. Control (**a**) shows the expression of CD34 protein at the lumen of the blood vessel and CAM treated with 3 μg leptin shows the expression at the capillary plexus or blood sinus area as coiled structure where endothelial cells pooled out (**b**, **d**) and also at those endothelial cell which is present at the lumen of the blood vessel (**c**, **e**). Images were recorded using light microscope. Arrow indicates the presence of CD34 protein, magnification 40X and bar is 50 μM

From our data it is clear that the angiogenic potential of HRL is maximum for 3 μg concentration enabling the formation of new blood vessels at the treated area. Activation of ECs is considered as one of the major step in angiogenic cascades especially during sprouting angiogenesis [27]. The result obtained from the study confirm the potential of HRL to activate ECs by identifying the presence of CD34 (activated endothelial cell marker) expression on ECs. The result indicates that the CD34 expression is observed at capillary plexus or blood sinus area in which ECs were pooled out along with angioblast cells (Fig. 7b) and the pattern of staining resembles more like coiled structures with elongated protrusions suggestive of the sprouting of ECs under HRL (Fig. 7d). Also the ECs present at the luminal side of the vessels shows higher staining for CD34 than those present in the control (Fig. 7a). Presence of these elongated ECs is suggestive of its enhanced proliferative capacity when treated with HRL which in turn lead to the formation of capillary like structure during the process of angiogenesis especially when sprouting angiogenesis occurs (Fig. 7c and e). In control, those ECs which were present at the lumen of the main vessel shows CD34 expression and no staining was observed at the blood sinus area (Fig. 7a). Thus it can be concluded that HRL has the ability to enhance EC activation which in turn can favour these cells to participate actively in angiogenesis.

### Angiogenic potential of human recombinant leptin mainly depends on the activation of VEGF A and MMP2 – protein expression

**Fig. 8:**
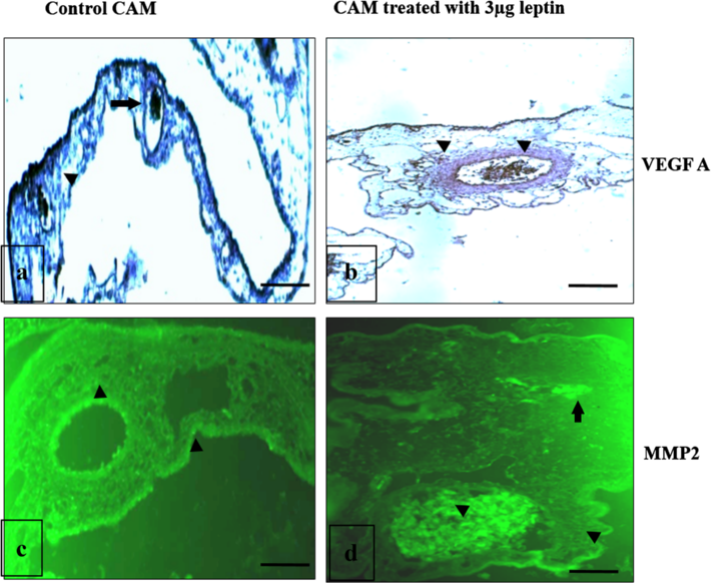
Human recombinant leptin induces the expression of VEGF165 and MMP2. Immunohistochemical images of CAM for VEGF165 and MMP2 after treated with 3 μg of HRL and control with 1X PBS for 72 h. CAM treated with 3 μg of HRL shows more expression of VEGF A at the lumen of the blood vessels (fig **b**) and MMP2 at the stroma (fig **d**) than control (fig **a** and **c**). Images are representative of 3 set of experiments and were recorded by light and BX51 Olympus fluorescence microscopes. Arrow indicates the presence of protein, magnification is 60X and bar 50 μM

Molecular profiling of angiogenic factors after treatment with HRL indicated that angiogenic potential of leptin mainly depends on the functional activation of VEGF A (VEGF 165) and MMP2. Hence we attempted to understand the same by scoring the protein level expression of VEGF A and MMP2 using immunohistochemical method. Results show that with 3 μg concentration of HRL the expression of VEGF A protein (Fig. 8b) is higher at the lumen of main vessels as well as at the surrounding small ones as an indicating enhanced ECs activation while control shows the expression only at the luminal surface of the main vessel (Fig. 8a). Expression of MMP2 protein after HRL treatment seems to be more concentrated at the vessel surface particularly at the lumen and also at the thin epithelial layer which contains peptidoglycan extra cellular matrix (Fig. 8d) and this is higher than that of control (Fig. 8c). Presence of increased level of MMP2 protein at the lumen of the vessel is suggestive of the active breakdown of extra cellular matrix in favour of ECs migration and proliferation, support the occurrence of sprouting angiogenesis after HRL treatment. Thus the expression pattern illustrates that HRL able can activate the pool of ECs in such a way that these cells can actively participate in the angiogenic cascade process. The results also demonstrates that 3 μg of HRL is capable of inducing neovascularization by increasing the degradation ECM which in turn allows the micro vascular endothelial cells to migrate, proliferate and differentiate. Presence of VEGF A at the endothelium also indicates that 3 μg of HRL could favour endothelial cell activation followed by sprouting, initiating new blood vessel formation.

## Discussion

Angiogenesis is defined as the formation of new blood vessels from existing ones which plays an important role in many physiological and pathological events [28]. Leptin, the Ob gene product is known to exert its biological activity through binding with it receptors termed Ob-R identified in hypothalamus and also in peripheral vascular tissue such as ECs [1, 29]. Reports from various *in vivo* analyses have suggested both angiogenic and anti angiogenic property of leptin [3, 10]. But no reports have yielded conclusion results. Reports have also suggested that this dual function of leptin depends on multiple factors such as dose, time of incubation, mode of administration and to certain extent the type of species [3, 4, 8–10, 30].

In this context we analysed the angiogenic potential of HRL using *in vivo* CAM assay. The main advantage of the assay is that it can be used as a rapid method of determining the angiogenic responses because it is considered as an intermediate step between a single model (cell culture) and a more complex system (mammalian model). The other important advantage is that CAM can be used to score the tissue responses towards the angiogenic activity of bio materials accurately and are similar to that of mammalian model responses [31] and also helps in the maintenances of the test materials at the site of administration. To overcome this technical problem we used gelatin sponges for the delivery of the chemical which can held and adhere firmly to the CAM surface with no or less inflammatory reactions [13, 32].

In the present work we found that *in ovo* administration of HRL is able to induce neovascularization at the treated area in a dose dependent manner. Among those analysed concentration (1, 3 and 5 μg) only 3 μg of HRL shows significant angiogenic response as observed the increase in vessel growth at an early incubation period of 48 h. The results are in accordance with the earlier research report where the effect was prominent even at 24 h of incubation [5]. But this shorter time period of incubation on CAM is considered as vasodilation period rather than compound effect [13]. Hence in the present study we performed the experiments for 72 h to analyse the angiogenic potential of HRL because at this time point newly formed vessels will become stabilized. Interestingly we found that at 72 h of incubation both 3 and 5 μg of HRL shows significant angiogenic effect indicating that the angiogenic potential of HRL not only depends on the concentration but also on the duration of incubation. Microscopic analysis of the growth pattern of the vessels implicates that 3 μg of leptin is able to induce more sprouting of new vessels from the existing one when compared to 5 μg concentration. Thus, HRL has the potential to induce neovascularization in a dose dependent manner and that 3 μg concentration could be more appropriate resulting in earlier and stable angiogenesis.

Placing any foreign material onto CAM can develop inflammatory reaction which can cause secondary vasoproliferative response leading to false positive conclusion [13]. To avoid this issue we have performed a thorough histological evaluation after adding HRL. Changes in the histological structure and altered cellular morphology of the treated area together indicated that HRL is able to initiate new blood vessel formation without local inflammation. HRL of 3 μg shows comparatively higher angiogenic responses by accelerating the formation of new vessel through sprouting from the major one. Also the angiogenic or antiangiogenic effect of compound can be effectively analysed by measuring the thickness of the CAM at the treated area as formation and growth of the new blood vessels will result in an increased tissue thickness at the stroma region. Any changes related to the growth at the stroma will push chorion and allantoic epithelium a part [15]. In our study we found that 3 μg of HRL is able to exert maximum angiogenic response at the stroma region very near to chorionic epithelium rather than mesodermal layer by means of forming capillary like tubes and structures leading to increased tissue thickness.

Results from rat corneal assay as also from various *in vitro* analyses showed that angiogenic effect of leptin is mediated by VEGF [33] and is supported by matrix remodelling [34]. Molecular profiling of VEGF and its isoforms such as VEGF165 and VEGF121 indicates that HRL induced angiogenesis mainly depends on the expression of VEGF165 rather than VEGF121. Increased mRNA level of MMPs especially MMP2 confirmed the major role of MMP2 in matrix remodelling during HRL induced angiogenesis and is in agreement with the earlier report [29, 34, 35]. Various reports have suggested that the physiological functions of leptin get initiated once it binds to its receptor Ob-R present in various cellular systems. During angiogenesis leptin is shown to exert its activity by binding with Ob-R receptor present on ECs followed by up regulation of the downstream signalling [29]. Here also it is possible that the angiogenic response of HRL on CAM is mediated by the receptor Ob-R considering the fact that human and chicken leptin receptor has 62 % of homology and the structural domains of the receptor are highly conserved in both [36]. The present data suggests that 3 μg of HRL has more angiogenic potential by inducing the increased mRNA level expression of VEGF165, VEGF121, bFGF2, MMP2 and MMP9 and also the protein level expression of VEGF165 and MMP2. Thus of HRL could up regulate the expression of VEGF165 and MMP2 to induce neovascularization resulting in the observed sprouting at the area of treatment.

Activation of EC is considered as one of the most important and preliminary step in the cascade of angiogenesis [28]. *In vitro* analysis suggested that leptin could initiate the activation of ECs to form tube like structures while inducing angiogenesis [29]. In this work we also analysed the potential effect of HRL on the activation of ECs by studying the immuno localization of CD34 expression on CAM ECs. Presence of more activated ECs at the blood sinus region along with coiled like structures formation of ECs at the sinus area together highly support the ability of HRL to activate ECs during angiogenesis. Presences of elongated ECs at the lumen of the vessel also indicates that 3 μg of HRL could be the optimum concentration that can initiate the formation of new capillary like structures which is remarkable at the first phase of sprouting angiogenic process.

## Conclusion

The present work demonstrates that HRL has the potential to induce neovascularization by means of sprouting at the treated area. Expression of CD34 on activated ECs indicates that HRL can initiate the activation ECs and could favour the formation of tube like structure from the pool of ECs. Increased tissue thickness and altered cellular morphology of CAM with HRL treatment supports dose dependent angiogenic ability of HRL. It was also observed that HRL induced angiogenesis mainly depends on the activation of VEGF165 and MMP2. Direct visualization and growth of newly formed vessels at the treated area demonstrates that the angiogenic ability especially sprouting effect of HRL depends on the concentration and time of incubation. It is found that 3 μg of HRL exhibits significant angiogenic response at 72 h of incubation. Altogether our findings suggest that HRL could be a useful angiogenic therapeutic molecule and can be more effectively used in the future especially for the treatment of ischemic disorders and wound healing which requires new vessel formation.
